# Key summary of German national treatment guidance for hospitalized COVID-19 patients: Key pharmacologic recommendations from a national German living guideline using an Evidence to Decision Framework (last updated 17.05.2021)

**DOI:** 10.1007/s15010-021-01645-2

**Published:** 2021-07-06

**Authors:** Jakob J. Malin, Christoph D. Spinner, Uwe Janssens, Tobias Welte, Steffen Weber-Carstens, Gereon Schälte, Petra Gastmeier, Florian Langer, Martin Wepler, Michael Westhoff, Michael Pfeifer, Klaus F. Rabe, Florian Hoffmann, Bernd W. Böttiger, Julia Weinmann-Menke, Alexander Kersten, Peter Berlit, Marcin Krawczyk, Wiebke Nehls, Falk Fichtner, Sven Laudi, Miriam Stegemann, Nicole Skoetz, Monika Nothacker, Gernot Marx, Christian Karagiannidis, Stefan Kluge

**Affiliations:** 1 Division of Infectious Diseases, Department I of Internal Medicine, University of Cologne, Cologne, Germany; 2 Department of Internal Medicine II, School of Medicine, Technical University of Munich, University Hospital Rechts Der Isar, Munich, Germany; 3 Medical Clinic and Medical Intensive Care Medicine, St Antonius Hospital, Eschweiler, Germany; 4 Department of Respiratory Medicine and German Centre of Lung Research (DZL), Hannover Medical School, Hannover, Germany; 5 Department of Anesthesiology and Operative Intensive Care Medicine (CCM/CVK), Charité-Universitätsmedizin Berlin, Berlin, Germany; 6 Department of Anaesthesiology, Medical Faculty Aachen, Rhenish Westphalian Technical University (RWTH), Aachen, Germany; 7 Institute of Hygiene and Environmental Medicine, Charité-University Medicine, Berlin, Germany; 8 Department of Hematology and Oncology, University Medical Center Hamburg-Eppendorf, Hamburg, Germany; 9 Department of Anesthesiology and Intensive Care Medicine, University Hospital Ulm, Ulm, Germany; 10 Department of Pneumology, Intensive Care and Sleep Medicine, Hemer Lung Clinic Centre of Pneumology and Thoracic Surgery, 58675 Hemer, Germany; 11 Department of Internal Medicine II, University Hospital Regensburg, Regensburg, Germany; 12 Department of Pneumology, Donaustauf Hospital, Donaustauf, Germany; 13 LungenClinic Grosshansdorf, Airway Research Centre North, German Centre for Lung Research, Grosshansdorf, Germany; 14 Department of Pediatrics, Dr. von Hauner Children’s Hospital, University Hospital, Ludwig-Maximilians-University Munich, Munich, Germany; 15 Department of Anesthesiology and Intensive Care Medicine, Medical Faculty and University Hospital Cologne, University of Cologne, Cologne, Germany; 16 Division of Nephrology, Department of Internal Medicine I, University Medical Center of the Johannes Gutenberg University, Mainz, Germany; 17 Department of Cardiology, Angiology and Intensive Care, Medical Faculty RWTH Aachen, Aachen, Germany; 18 Germany German Society of Neurology, Berlin, Germany; 19 Department of Medicine II, Saarland University Medical Center, Saarland University, Homburg, Germany; 20 Department of Palliative Care and Geriatric Medicine, Helios Clinic Emil Von Behring, Berlin, Germany; 21 Department of Anesthesiology and Intensive Care, University Hospital of Leipzig, Leipzig, Germany; 22 Department of Infectious Diseases and Respiratory Medicine, Charité-Universitätsmedizin Berlin, Berlin, Germany; 23 Evidence-Based Oncology, Department I of Internal Medicine and Center for Integrated Oncology Aachen Bonn Cologne Dusseldorf, Faculty of Medicine, University Hospital Cologne, University of Cologne, Cologne, Germany; 24 Institute for Medical Knowledge Management, Association of the Scientific Medical Societies (AWMF), Universität Marburg, Marburg, Germany; 25 Department of Intensive Care Medicine and Intermediate Care, Medical Faculty, University Hospital RWTH Aachen, Aachen, Germany; 26 Department of Pneumology and Critical Care Medicine, Cologne-Merheim Hospital, ARDS and ECMO Centre, Kliniken Der Stadt Köln, Witten/Herdecke University Hospital, Cologne, Germany; 27 Department of Intensive Care, University Medical Center Hamburg-Eppendorf, Hamburg, Germany

**Keywords:** COVID-19, SARS-CoV-2, Living guideline, Living meta-analysis

## Abstract

**Purpose:**

This executive summary of a national living guideline aims to provide rapid evidence based recommendations on the role of drug interventions in the treatment of hospitalized patients with COVID-19.

**Methods:**

The guideline makes use of a systematic assessment and decision process using an evidence to decision framework (GRADE) as recommended standard WHO (2021). Recommendations are consented by an interdisciplinary panel. Evidence analysis and interpretation is supported by the CEOsys project providing extensive literature searches and living (meta-) analyses. For this executive summary, selected key recommendations on drug therapy are presented including the quality of the evidence and rationale for the level of recommendation.

**Results:**

The guideline contains 11 key recommendations for COVID-19 drug therapy, eight of which are based on systematic review and/or meta-analysis, while three recommendations represent consensus expert opinion. Based on current evidence, the panel makes strong recommendations for corticosteroids (WHO scale 5–9) and prophylactic anticoagulation (all hospitalized patients with COVID-19) as standard of care. Intensified anticoagulation may be considered for patients with additional risk factors for venous thromboembolisms (VTE) and a low bleeding risk. The IL-6 antagonist tocilizumab may be added in case of high supplemental oxygen requirement and progressive disease (WHO scale 5–6). Treatment with nMABs may be considered for selected inpatients with an early SARS-CoV-2 infection that are not hospitalized for COVID-19. Convalescent plasma, azithromycin, ivermectin or vitamin D_3_ should not be used in COVID-19 routine care.

**Conclusion:**

For COVID-19 drug therapy, there are several options that are sufficiently supported by evidence. The living guidance will be updated as new evidence emerges.

## Background

The ongoing COVID-19 pandemic causes an enormous pace and volume of research output of varying quality and reliability which is challenging for clinical decision making. To provide a clinical treatment guideline that includes a profound evaluation of rapidly changing evidence, the scientific medical societies conduct a living treatment guideline for hospitalized patients with COVID-19 moderated by the Association of the Scientific Medical Societies in Germany (AWMF) in collaboration with the COVID-19 Evidence Ecosystem Project (CEOsys). CEOsys is an association of 20 German university hospitals whose goal is to compile, summarize, and assess the quality of study results within a structured approach and meta-analysis. Evidence profiles of treatment strategies provided by CEOsys form the basis for a structured decision process using the GRADE (Grading of recommendations assessment, development and evaluation) Evidence to Decision Framework [2] with predefined outcomes in compliance with the WHO Handbook for Guideline Development [1]. Here, we present key recommendations for the most pertinent drug interventions that derived from this national living treatment guideline (last updated 17.05.2021). A full length guideline and evidence report is available in German language [3]. The following assessments are subject to constant evaluation and will be re-evaluated, revised or supplemented at short intervals.

## Methods

### Aims of the guideline

The AMWF S3 guideline aims to provide a comprehensive overview of evidence based recommendations on therapeutic strategies for hospitalized patients with COVID-19. The guideline addresses physicians involved in the inpatient care of patients with COVID-19 but is also intended to provide information for individuals and organizations directly or indirectly involved in this topic. This compendium highlights the key pharmacologic treatment recommendations of the guideline that are most important for clinical care.

### Determination of guideline questions and relevant outcomes

The guideline group comprises actually 23 delegates from 15 participating scientific medical societies and organizations, as well as a patient representative (appendix 1).The guideline developed from an intensive care guideline based on an expert consensus in March 2020 [4] to an interdisciplinary evidence and consensus based guideline for all in-patients with COVID-19 with definition of complementary clinically relevant questions and formal consensus conferences starting in October 2020 [5]. A systematic evidence analysis was first integrated in February 2021 and now updated in Mai 2021. The following outcomes were defined as clinically relevant: 28-day mortality (up to); improvement/worsening of clinical status up to 28 days (including need for respiratory support, ventilator free days); weaning/liberation from mechanical ventilation, increase in WHO clinical progression scale (WHO scale) [6], adverse events (any grade), severe adverse events, need for dialysis (up to 28 days), neurological function/functional independence, duration of hospitalization, time to discharge, time to symptom resolution, viral clearance and specific outcomes for the assessment of prophylactic or intermediate anticoagulation (major bleeding or thrombotic events).

### Systematic literature search and processing of available evidence

Clinical questions on treatment of COVID-19 were assessed by a structured literature search and analysis supported by CEOSys (appendix 1) using the Patient-Intervention-Comparison-Outcome (PICO) format. Detailed search strategies are displayed in the full length evidence report [3]. Results of the weekly searches were screened in duplicate. Additionally, the guideline group performed literature searches on Medline database to ensure that recently published study results of relevance are included. Evidence for medical therapeutic strategies derived from (peer-reviewed) randomized clinical trials was extracted and bias assessed using Risk of bias 2.0. A meta-analysis was performed for all PICO questions where sufficient evidence and comparable outcomes were available. The quality of the evidence was assessed using the GRADE (Grading of recommendations assessment, development, and evaluation) methodology on predefined outcomes described earlier. The GRADE approach provides a standardized evaluation of the quality of the evidence which can finally be classified as high, moderate, low or very low [2]. Comprehensive evidence profiles for the different therapeutic strategies were created inside MAGICapp (https://app.magicapp.org), a digital platform and evidence ecosystem committed to support rapid recommendations and living guidelines during the COVID-19 pandemic [7]. Online manuscripts of clinical trials that were published as pre-prints were noticed but excluded from the systematic evaluation. Due to insufficient clinical data or COVID-19 specific data, key recommendations 2.4 (SARS-CoV-2 neutralizing monoclonal antibodies), 3.1, and 3.2 (anticoagulation) represent expert opinions based on available (partly observational or retrospective) evidence.

### Preparation of recommendations

All members of the panel had access to the evidence profiles on the MAGICapp platform [7] in preparation for the consensus conferences. During two consensus conferences, evidence profiles were presented by a dedicated editor of the CEOsys group responsible for the respective evidence profile before results were discussed in plenary under neutral moderation. Based on the evidence profiles a structured evaluation of the respective treatment strategy was performed that included an appraisal of the following criteria by the guideline group: benefit and harm balance, quality of the evidence, patient preferences, resources, equity, acceptability, and feasibility. Based on the evidence profile and overall assessment, recommendations were written and graded according to the AWMF standards (↑↑ = Strong recommendation (should/should not), ↑ = Recommendation (ought/ought not), ↔  = Recommendation open (may be considered)) [8]. For each recommendation one delegate per medical society and organization had to vote (agree, disagree, abstention). Guideline group members with conflicts of interest were excluded from the respective voting. A strong consensus (agreement > 95%) was achieved for 9 out of 11 key recommendations while a consensus (agreement > 75%) was achieved for two of them. For each recommendation, background information was summarized by working groups within the guideline group to describe available evidence and rationale for the chosen grading to make the decision process as transparent as possible. Contents were finally reviewed by all members of the guideline group and officially validated by the participating medical societies and organizations as well as the AWMF Task Force COVID-19 and experts from the Robert Koch Institute.

### Selection of key recommendations

For this summary, key recommendations of the guideline were selected that represent the most pertinent drug interventions for hospitalized patients with COVID-19. Background information and rationales for the respective recommendation are presented in a summarized format. A full length version of the guideline (in German) and a comprehensive evidence report is available at: https://www.awmf.org/leitlinien/detail/ll/113-001LG.html [3].

## Key recommendations

### 1. Immunomodulatory therapy

#### 1.1 Corticosteroids (meta-analysis)

**Patients with severe (SpO**_**2**_ **< 90%, respiratory rate > 30/min) or critical COVID-19 (ARDS, sepsis, ventilation, vasopressor administration) should be treated with dexamethasone ⇑⇑**

[Quality of evidence: 28d mortality: moderate, adverse events: low; strong consensus].

**Evidence**: Six RCTs with a total 7595 randomized patients were included [9–14]. For the clinically most relevant endpoint, a reduction in mortality by day 28, the meta-analysis of five RCTs that reported on this outcome with a total of 7527 hospitalized COVID-19 patients (WHO scale 4–9) demonstrated an overall reduction in mortality by day 28 with an absolute risk reduction of 2.8% in hospitalized patients treated with corticosteroids (moderate quality evidence). There was an apparent trend towards a higher effect with increased disease severity. The clearest evidence existed for dexamethasone administered at a dose of 6 mg daily. In the largest RCT on dexamethasone, an absolute mortality reduction of 12% was demonstrated in patients that require invasive ventilation (WHO scale 7–9), and 3% for patients requiring oxygen supplementation but no respiratory support (WHO scale 5–6) [9]. In contrast, dexamethasone showed no beneficial effects in hospitalized patients without any oxygen requirement (WHO scale 4). Actually, the meta-analysis showed a numerically higher 28-day mortality for this sub group when treated with dexamethasone (statistically significant subgroup difference) [3]. The CODEX trial did not demonstrate a significant reduction in 28-day mortality for invasively ventilated patients (secondary endpoint analysis), but a beneficial effect on the number of ventilator-free days (primary endpoint) [10]. The safety and tolerability of corticosteroids in hospitalized patients with COVID-19 was generally good in clinical trials (low quality evidence). The currently available data do not suggest an increased susceptibility for secondary (bacterial) infections due to corticosteroid regimens used in COVID-19 trials.

**Rationale for the level of recommendation**: Given the beneficial effects on mortality in patients requiring supplemental oxygen or invasive ventilation, a favorable safety profile and its wide availability, the guideline group strongly recommends the use of dexamethasone in these patient groups. The recommended dose is 6 mg dexamethasone p.o./i.v. daily for 10 days. In justified cases, another systemic glucocorticoid (e.g., hydrocortisone 50 mg i.v. every 8 h) can be used as an alternative.

#### 1.2 Tocilizumab (meta-analysis)

**Tocilizumab may be administered to patients with high-dose supplemental oxygen progressing to severe COVID-19 (WHO scale 5–6). Tocilizumab should not be used in patients with no or low supplemental oxygen requirement and in patients on invasive ventilation ⇔**

[Quality of evidence: 28d mortality: moderate, adverse events: low; strong consensus].

**Evidence**: Nine RCTs with a total of 6481 patients were included to evaluate tocilizumab [15–23]. Most patients additionally received concomitant therapy with dexamethasone as current standard of care. The meta-analysis showed a statistically significant effect on the clinically relevant endpoints mortality (36 fewer cases per 1000, 95% CI 57–12 fewer) and progression to invasive (mechanical) ventilation (44 fewer cases per 1000, 95% CI 63–25 fewer; moderate quality of evidence). Overall, a clinically relevant benefit can be concluded for oxygenated patients with progressive disease (WHO scale 5–6), but not for patients on invasive ventilation (for more than 24 h). The RECOVERY trial with 2022 patients included in the tocilizumab arm (intention-to-treat population), had a major impact on the overall effect in the meta-analysis [22]. In this trial, C-reactive protein (CRP) elevation > 75 mg/L was applied as an inclusion criterion reflecting a surrogate marker of systemic inflammation. Safety and tolerability of tocilizumab was favorable in the trials that provided detailed reporting of safety data, with no evidence of increased rates of (serious) adverse events. However, safety data from the RECOVERY trial seems to be incompletely reported (low quality of evidence).

**Rationale for the level of recommendation**: Based on the statistically significant but small net effect of tocilizumab, an open recommendation (may be used) was made for the use of this agent in combination with corticosteroids (SOC) for patients requiring oxygen supplementation and progressive disease. Patients should demonstrate evidence of a systemic inflammation (e.g., markedly elevated CRP) and increased oxygen demand. For patients already requiring invasive ventilation for a period longer than 24 h, the guideline group found no evidence for a clinical benefit of tocilizumab. Tocilizumab should generally not be used in patients with confirmed intolerance to tocilizumab or with active bacterial or fungal infection. Currently, no data are available on the safety of tocilizumab in pregnancy. Dosing of tocilizumab is based on body weight (> 90 kg: 800 mg; ≤ 90 kg: 600 mg; ≤ 65 kg: 400 mg; ≤ 40 kg: 8 mg/kgKG) as a single intravenous dose. Due to lack of comparative study data no recommendation for repeated administration can be made. Evaluating the clinical use of tocilizumab the guideline group took into account existing qualitative uncertainties of the evidence [3] and relatively high costs with undefined reimbursement regulations.

### 2. Antiviral therapy

#### 2.1 Remdesivir (meta-analysis)

**For hospitalized, non-ventilated patients with COVID-19 pneumonia and oxygen demand, no fundamental recommendation can be made neither for nor against therapy with remdesivir**

[Quality of evidence: 28d mortality: moderate, adverse events: low; consensus].

**Evidence**: A total of four RCTs were included in the assessment [24–27]. In a randomized, double-blind trial of 1062 hospitalized patients (ACTT-1), a 10-day course of remdesivir reduced the time to recovery from a median of 15 to 10 days compared to placebo [risk ratio for recovery, 1.29; 95% CI, 1.12–1.49; *P* < 0.001]. There was a numerical reduction in all-cause mortality at day 29 (11.4% vs. 15.22%, hazard ratio, 0.73; 95% CI, 0.52–1.03) [24]. Comparing the efficacy of a 5 vs. 10–day course of remdesivir, 10 days of remdesivir was not superior for patients with moderate to severe COVID-19 (WHO scale 5–6) and radiographic evidence of pulmonary infiltrates [28]. A smaller RCT of 237 patients failed to demonstrate a beneficial effect of remdesivir on time to clinical improvement [27], and results of another trial remained inconclusive [26]. In the WHO SOLIDARITY trial, 2750 patients received remdesivir. No benefit was found regarding clinical endpoints (mortality, initiation of ventilation, duration of hospitalization) [25]. In the meta-analysis based on 7142 patients, no significant effect of remdesivir on 28-day mortality (moderate quality of evidence) or other predefined clinical endpoints was found (very low to moderate quality of evidence). Subgroup analyses were hampered by the heterogeneity of the trial protocols and outcome definitions, so that no clear conclusion can be made on possible effects upon specific patient populations. The safety and tolerability of remdesivir in clinical trials was good. Fewer severe adverse events (SAE) were reported in remdesivir treated groups compared to placebo (relative risk 0.75; CI 95% 0.63–0.90; low quality evidence).

**Rationale for the level of recommendation**: Due to persisting uncertainties regarding a potential benefit of remdesivir in hospitalized, non-ventilated patients, and relevant costs that must be considered, no recommendation was made. The World Health Organization (WHO) has issued a conditional recommendation against the use of remdesivir in hospitalized patients, due to insufficient evidence for improved survival and other clinical outcomes [29]. The use of remdesivir in ventilated patients is not appropriate due to lack of any clinical benefit in this population. The guideline group considers a potential benefit for specific subgroups and/or on clinical endpoints that were not included in the meta-analysis, especially time to recovery (primary endpoint of the ACTT-1 study). In the light of its favorable safety profile and tolerability, no recommendation against the use of remdesivir was established within the structured guideline process. If treatment with remdesivir is considered by clinicians, the dose should be 200 mg i.v. on day 1, followed by 100 mg daily for 5 days. A prolonged therapy for up to 10 days may be considered if the effect is considered insufficient. Daily monitoring of liver and renal function parameters is recommended; remdesivir should not be administered in patients with a GFR < 30 ml/min. Regarding the optimal timing of antiviral therapy, there are theoretical considerations and indications that treatment initiation as early as possible in the course of the disease might be favorable [30].

#### 2.2 Convalescent plasma (meta-analysis)

**Convalescent plasma should not be used in hospitalized patients with COVID-19. No recommendation can be made upon specific subgroups based on current evidence ⇓⇓**

[Quality of evidence: 28d mortality: moderate, adverse events: low; strong consensus].

**Evidence**: Four randomized controlled trials with a total of 931 patients [31–34] built up the evidence base for this recommendation. Based on the meta-analysis, no clinical benefit was identified regarding 28-day mortality and clinical status (moderate quality of evidence). Concurrently, there was a trend towards an increased occurrence of adverse events and serious adverse events in groups treated with convalescent plasma (very low to low quality of evidence). There is an inherent risk of transfusion-related reactions that must be taken into account when administering plasma preparations.

**Rationale for the level of recommendation**: In weighing the risks and benefits of convalescent plasma, the guideline group strongly recommends against its use in hospitalized patients with COVID-19. Besides the lack of evidence supporting a clinical benefit, the panel included additional considerations for the recommendation such as limited resources for logistics of donation, administration, processing, storage and distribution as well as financial aspects. Production of convalescent plasma requires sufficient donor availability. Considering these aspects, a widespread availability of this treatment seems unlikely.

#### 2.3 SARS-CoV-2 neutralizing monoclonal antibodies (nMABs)

##### 2.3.1 Bamlanivimab monotherapy (systematic analysis)

**The SARS-COV-2 neutralizing monoclonal antibody (nMAB) bamlanivimab should not be used to treat patients hospitalized due to moderate to severe COVID-19 (WHO scale 4–6) ⇓**

[Quality of evidence: 28d mortality: moderate, adverse events: low; strong consensus].

**Evidence**: One RCT of 314 hospitalized patients was available. A clinical benefit of bamlanivimab in patients hospitalized for moderate to severe COVID-19 (WHO scale 4–6) was not shown in the ACTIV3/TICO trial (low to moderate quality evidence; [35]). In the intervention group, there was a numerically higher incidence of hemodialysis (13/1000 vs. 0/1000), delirium (26/1000 vs. 7/1000), and grade 3–4 adverse events (227/1000 vs. 179/1000), so that potentially harmful effects cannot be completely excluded (low quality of evidence).

**Rationale for the level of recommendation**: Based on available evidence, the guideline group strongly recommends against bamlanivimab monotherapy in patients hospitalized for COVID-19. There is currently insufficient evidence on combinational therapy with multiple nMABs, so no recommendation can be formulated.

##### 2.3.2 SARS-CoV-2 neutralizing monoclonal antibodies in nosocomial infection

**In early SARS-CoV-2-infected hospitalized patients without respiratory COVID-19 symptoms (< 72 h after first positive NAAT and/or < 7 days since symptom onset), with at least one risk factor for a severe course of disease, SARS-CoV-2 specific monoclonal antibodies may be administered ⇔**

[Expert opinion; consensus].

**Evidence**: Currently, there are insufficient data to evaluate the use of neutralizing SARS-CoV-2 monoclonal antibodies (nMABs) in hospitalized patients. Results from outpatient trials cannot be adequately extrapolated to (often nosocomial) infected inpatients not hospitalized for COVID-19. This recommendation, therefore, represents consented expert opinions considering available evidence on the efficacy and tolerability of nMABs in general as well as personal off-label use experience.

**Rationale for the level of recommendation**: Interim analyses of ongoing phase II trials indicate that early (≤ 7 days after symptom onset) administration of combination therapies with bamlanivimab and etesevimab, as well as casirivimab and imdevimab might be beneficial in outpatients with mild to moderate COVID-19 disease (WHO scale 1–3) and at least one risk factor for disease progression. Early administration of nMABs resulted in a significant reduction in viral load, and (numerical) reduction in hospitalization rate or subsequent physician contacts [36–38]. However, rates for hospitalization or physician contacts were generally low in the patient population included in published trial data. A conclusive assessment on clinical endpoints is currently not possible on this basis. Known risk factors for a severe course of COVID-19 include: Age > 65 years, obesity with body mass index (BMI) > 35, immunosuppression, chronic renal failure, chronic lung disease such as COPD, or trisomy 21. The guideline group emphasizes the risk for a progressive (nosocomial) SARS-CoV-2 infection in patients hospitalized for an indication other than COVID-19. An additional argument is the opportunity of very rapid nMAB administration early in the course of infection due to the hospitalized state. Ideally, nMABs should be given ≤ 72 h after the initial SARS-CoV-2 detection (by PCR or other Nucleic Acid Amplification Technology, NAAT) and (if appropriate) ≤ 7 days after symptom onset. When considering therapy with nMABs, the prevalence of circulating resistant variants must be taken into account. In the presence of an E484K mutation (B.1.351 or P1 variant), bamlanivimab (± etesevimab) cannot expected to be effective [39]. As nMAB-monotherapy has been associated with the emergence of escape mutations, experts discuss the preferential use of combination drugs, especially in immunosuppressed patients.

### 3. Anticoagulation

#### 3.1 Pharmacological thromboprophylaxis

**Hospitalized patients with COVID-19 should receive prophylactic anticoagulation with standard dose low molecular weight heparin (LMWH) in the absence of contraindications. Alternatively, fondaparinux may be used **[40]** ⇑⇑**

[Expert opinion; strong consensus].

**Evidence**: Thromboembolic events are common complications of moderate to severe COVID-19 predominantly affecting the venous system and to a lesser extend arterial vessels [41, 42]. There are no specific data or insights regarding pharmacological thromboprophylaxis in COVID-19. The recommendation is, therefore, based on the AWMF S3 guideline ‘Prophylaxis of Venous Thromboembolism’ that addresses the general hospitalized patient population [40]. The benefit of prophylactic anticoagulation, preferably with LMWH, in hospitalized patients has been prospectively investigated in several randomized trials and demonstrated high efficacy in reducing the risk of venous thromboembolisms (VTE) without significantly increasing the risk of major bleeding [43–45].

**Rationale for the level of recommendation**: Based on a large body of evidence for the use of pharmacological thromboprophylaxis in hospitalized patients the guideline group strongly recommends its use for all hospitalized patients with COVID-19, preferentially with low-molecular-weight heparin (LMWH) at a dosage approved for the high-risk setting. Alternatively, e.g., in case of heparin intolerance, previous heparin-induced thrombocytopenia (HIT) or other specific contraindications, fondaparinux can be used.

#### 3.2 Intensified anticoagulation

In hospitalized patients with COVID-19 and additional risk factors for venous thromboembolism (VTE), intensified anticoagulation* may be used if the bleeding risk is low. Additional risk factors for VTE include obesity (BMI > 35 kg/m^**2**^**), previous VTE, known thrombophilia, intensive care treatment and highly increased D-dimers (> 2–3 mg/l) **[46]** ⇔**

[Expert opinion; strong consensus].

*For example: intermediate, half-therapeutic dose of LMWH. No universal definition exists for intermediate dose of unfractionated heparin (UFH). A dose of 250 IU/kg over 24 h and/or a 1.5-fold prolongation of the baseline aPTT value can serve as guidance.

**Evidence**: Observational data suggest that prophylactic dose anticoagulation may not be sufficiently effective for VTE prophylaxis in patients with COVID-19, especially those that require ICU treatment [47, 48]. In a systematic meta-analysis of (mostly retrospective) observational studies, the rate of VTE was numerically lower when using intermediate-dose anticoagulation compared to prophylactic anticoagulation [46]. In a prospective randomized trial of 600 intensive care patients, intermediate-dose NMH (enoxaparin 1 mg/kg daily) showed no advantage over prophylactic NMH (enoxaparin 40 mg daily) regarding the combined efficacy endpoint of venous or arterial thromboembolism, need for ECMO therapy, and 30-day mortality (event rate 45.7% vs. 44.1%; odds ratio 1.06; 95% CI 0.76–1.48; *P* = 0.70). While VTE rates were similar in both groups (3.3% vs. 3.5%), intermediate-dose anticoagulation led to a numerically higher rate of clinically relevant bleedings (6.2% vs. 3.1%.; OR 2.02; 95% CI 0.89–4.61; *P* = 0.08) [49]. To date, no specific RCT on intermediate-dose anticoagulation in hospitalized patients with non-critical COVID-19 has been published but one is now under peer-review [50].

**Rationale for the level of recommendation**: Due to the risk of thromboembolic complications in patients with COVID-19, especially in case of severe disease requiring intensive care therapy, the panel suggests that intensified anticoagulation, e.g., with an intermediate, half-therapeutic dose of NMH, may be used in patients with additional risk factors for VTE and a low bleeding risk. Risk factors for bleeding include severe hepatic or renal impairment, thrombocytopenia, previous bleeding, antiplatelet therapy or recent surgery. Due to the potential harms of intensified anticoagulation and an unclear net benefit, routine use of intermediate-dose anticoagulation in patients with COVID-19 is not recommended.

### 4. Miscellaneous

#### 4.1 Azithromycin

**Azithromycin should not be administered to hospitalized COVID-19 patients as antiviral therapy ⇓⇓**

[Quality of evidence 28d mortality: high, adverse events: moderate; strong consensus].

**Evidence**: Based on in vitro antiviral effects, azithromycin has been evaluated as therapeutic agent for the treatment of COVID-19 in four randomized controlled trials including a total of 8759 patients [51–54]. Within the largest trial RECOVERY, more than 7700 hospitalized patients (94% did not require invasive ventilation), were randomized to receive azithromycin in addition to SOC therapy [51]. The meta-analysis revealed no benefit of azithromycin with respect to patient-relevant outcomes such as mortality, clinical improvement, need for invasive ventilation, and length of hospitalization (moderate to high quality of evidence). Adverse events and secondary infections were slightly increased in groups treated with azithromycin compared to placebo (low to moderate quality of evidence) while severe adverse events and cardiac arrhythmias were not increased.

**Rationale for the level of recommendation**: A beneficial effect of azithromycin could not be proven but the guideline group cannot exclude potential harms of this treatment for patients with COVID-19 (without additional bacterial infection). A negative impact of increased macrolide on the spread of antibiotic-resistant bacteria has not been systematically investigated in the context of COVID-19 but seems possible. It is important to note that azithromycin was assessed for (antiviral) effects on COVID-19, but not for its (known) antibacterial activity. For cases of suspected or proven bacterial co-infection we refer to comprehensive and specific guidelines [3, 5, 55, 56].

#### 4.2 Ivermectin

**Ivermectin should not be used to treat hospitalized patients with COVID-19 ⇓⇓**

[Quality of evidence time to virus elimination: very low, length of hospital stay: very low; strong consensus].

**Evidence**: Numerous preprints are available online reporting solely on viral load reduction rather than clinically relevant endpoints or with significant methodological issues and a high risk of bias (exemplary [57–59]). At the time of consensus for this recommendation, February 2021, only one peer-reviewed publication of a randomized controlled trial with a total of 72 patients was available for consideration [60]. In this trial, hospitalized patients without oxygen supplementation (WHO scale 4) were treated with ivermectin, ivermectin in combination with doxycycline, or with placebo. Based on the results the authors suggest a faster viral elimination in consecutive nasopharyngeal swabs of patients treated with ivermectin in monotherapy (low quality evidence). However, there was no apparent effect on clinical endpoints such as symptoms or duration of hospitalization and results do not support any conclusion regarding mortality or improvement of clinical status.

**Rationale for the level of recommendation**: Considering potential toxic effects of ivermectin as well as interactions with other drugs, the guideline group strongly recommends against its use in patients with COVID-19. Besides the absence of a proven benefit, the appraisal included basic pharmacokinetic considerations as tissue concentrations achievable with oral administration of ivermectin seems to be far below the half maximal inhibitory concentration in vitro [61].

#### 4.3 Vitamin D_3_

**Vitamin D**_3_ **should not be used to treat hospitalized patients with COVID-19 ⇓⇓**

[Quality of evidence: mortality very low; strong consensus].

**Evidence**: Due to the lack of randomized controlled trials (February 2021) only one study with a total of 76 patients could be included into the assessment [62]. In this clinical trial, administration of vitamin D3 demonstrated no benefit on patient-relevant outcomes (very low quality of evidence). Decreases in serum vitamin D_3_ are frequently observed in severely ill patients requiring intensive care treatment and may correlate with disease severity [63]. In analogy to other serious infections decreased serum vitamin D_3_ levels seem to be a relatively common phenomenon in COVID-19. This observation does not indicate a causal relationship and does not justify vitamin D3 supplementation in this setting. After completion of the primary evidence analysis, another study was published [64]. In this randomized controlled trial, vitamin D3 was administered to 240 hospitalized patients with COVID-19. No benefit was found with respect to clinical endpoints (duration of hospitalization, mortality, admission to the intensive care unit, initiation of ventilation).

**Rationale for the level of recommendation**: Considering available evidence, the guideline group strongly recommends against the use of vitamin D3 to treat hospitalized patients with COVID-19. Besides the lack of evidence, the guideline group took into account the generally widespread availability of vitamin D_3_ and low costs, the intention to avoid wrong incentives for self-medication and potential adverse effects that can result from overdosing. Due to the lack of therapeutic consequences, routine determination of serum vitamin D_3_ in COVID-19 is also not recommended which is in line with guidelines released by the German Nutrition Society [65].

## 5. Combinations of drug therapies

Despite theoretical considerations that combinational therapy might improve efficacy of COVID-19 treatment regimens [66], only few data are available. From the final ACTT-1 publication, a post-hoc analysis of a subgroup treated with remdesivir and steroids suggested a potential additive effect [24]. Further controlled trial data are lacking to date. The ACTT-2 study of 1033 patients showed that the combination of the Janus kinase inhibitor baricitinib with remdesivir was beneficial compared with remdesivir alone in terms of a 7 vs. 8-day median time to recovery and 30% higher probability of clinical recovery at day 15 [67]. A statistically significant mortality benefit was not shown. To date, there are no published peer-reviewed data from randomized controlled trials on the use of baricitinib compared to corticosteroids.

## Summary of key recommendations

Reliable evidence for clinical efficacy in hospitalized patients with moderate to severe COVID-19 is available for dexamethasone. The use of dexamethasone in patients requiring oxygen (WHO scale 5) and patients with severe/critical disease requiring ventilation (WHO 6–9) significantly reduces mortality (moderate quality of evidence). The interleukin-6 antagonist tocilizumab demonstrated a statistically significant but low absolute effect on mortality and progression to invasive ventilation in our meta-analysis for patients with moderate to severe COVID-19 (WHO 5–6, low to moderate quality of evidence). The guideline panel recommends to consider tocilizumab for patients with higher level of supplemental oxygen requirement and progressive disease but not for those with more than 24 h of mechanical ventilation. Evidence regarding antiviral treatment with remdesivir is conflicting and currently no fundamental recommendation is made. The use of remdesivir in patients with invasive ventilation or without oxygen supplementation in not reasonable based on current clinical evidence. If remdesivir is used in patients with oxygen supplementation, an early treatment and 5-day course of remdesivir seems the most rational approach. The use of pharmacological thromboprophylaxis is strongly recommended for all patients hospitalized with COVID-19 while an intensified anticoagulation may be considered in patients with additional risk factors for venous thromboembolisms (VTE) and a low bleeding risk (expert opinion). In the setting of SARS-CoV-2 infection and hospitalization due to other indications than COVID-19 and/or nosocomial SARS-CoV-2 infection, an early treatment (< 72 h after first positive PCR and/or < 7 days since symptom onset) with SARS-CoV-2 neutralizing monoclonal antibodies (nMABs) can be considered as it might reduce the risk of disease progression in patients with at least one risk factor for severe COVID-19 (expert opinion). Other agents cannot be recommended for use outside clinical trials and eligible clinical settings (Table 1).

**Table 1:** Summarizing table of key pharmacologic recommendations

Drug treatment	Recommendation^a^	WHO scale^b^	Evidence (sample size^c^)	Quality of evidence
1. Immunomodulators				
1.1 Corticosteroids	⇑⇑	5–9	6 RCT (*N* = 7595)	Moderate
1.2 Tocilizumab (anti-IL6)	⇔	(5-)6*	9 RCT (*N* = 6481)	Low to moderate
2. Antivirals				
2.1 Remdesivir	None	N/A	4 RCT (*N* = 7142)	Low to moderate
2.2 Convalescent plasma	⇓⇓	4–9	4 RCT (*N* = 931)	Low to moderate
2.3.1 Bamlanivimab (nMAB)	⇓	4–9	1 RCT (*N* = 314)	Low
2.3.2 nMABs for early nosocomial infection	⇔	(1–2)^#^	*Expert opinion*	*N/A*
3. Anticoagulation				
3.1 Pharmacological thromboprophylaxis	⇑⇑	4–9	*Expert opinion*	*N/A*
3.2 Intensified anticoagulation	⇔	4–9	*Expert opinion*	*N/A*
4. Miscellaneous				
4.1 Azithromycin	⇓⇓	4–9	4 RCT (*N* = 8759)	Moderate to high
4.2 Ivermectin	⇓⇓	4–9	1 RCT (*N* = 72)	Very low
4.3 Vitamin D_3_	⇓⇓	4–9	1 RCT (*N* = 76)	Very low

*nMABs* SARS-coV-2 neutralizing monoclonal antibodies; *RCT* randomized controlled trial

^a^Grading of recommendations using the GRADE approach [2]:

⇑⇑/⇓⇓ Strong recommendation for/against use (should/should not)

⇑/⇓ Weak recommendation for/against use (ought/ought not)

⇔ Open recommendation (may be considered)

^b^Corresponding WHO COVID-19 clinical progression scale [6] for the respective recommendation

^c^Including placebo group and randomized (intention to treat) population

*Not recommended for patients with low supplemental oxygen requirement (e.g., < 6L O_2_/min via nasal canula). Might be considered for patients with ≤ 24 h of invasive ventilation (WHO scale 7)

^#^Hospitalization due to indication other than COVID-19 and/or nosocomial infection

## Data Availability

Full length reports including evidence assessments based on published data are publicly available on the website of the Association of the Scientific Medical Societies in Germany (AMWF): https://www.awmf.org/leitlinien/detail/ll/113-001.html.

## Appendix 1

See Tables 2, 3, 4.

**Table 2:** Guideline coordination

Dr. Monika Nothacker	Association of the Scientific Medical Societies in Germany (AMWF)
Prof. Dr. Stefan Kluge	German Society for Medical Intensive Care Medicine and Emergency Medicine (DGIIN)

**Table 3:** Guideline group: delegates and represented German medical societies

Medical society	Delegate
German Society of Neurology (DGN)	Prof. Dr. Peter Berlit
German Resuscitation Council (GRC)	Prof. Dr. Bernd W. Böttiger
German Society of Hygiene and Microbiology (DGHM)	Prof. Dr. Petra Gastmeier
Patient representative	Reiner Haase
German Society of Pediatrics and Adolescent Medicine (DGKJ)	PD Dr. Florian Hoffmann
German Interdisciplinary Association for Intensive care medicine (DIVI)*	Prof. Dr. Uwe Janssens
German Society for Medical Intensive Care Medicine and Emergency Medicine (DGIIN)*	Prof. Dr. Christian Karagiannidis
German Cardiac Society (DGK)	Dr. Alexander Kersten
German Society for Medical Intensive Care Medicine and Emergency Medicine (DGIIN)*	Prof. Dr. Stefan Kluge (coordinating author)
German Society for Gastroenterology, Digestive and Metabolic Diseases (DGVS)	Dr. Marcin Krawczyk
Society for Thrombosis and Hemostasis Research (GTH)	Prof. Dr. Florian Langer
German Society of Infectious Diseases (DGI)*	Dr. Jakob J. Malin, MSc
German Society of Anaesthesiology and Intensive Care Medicine (DGAI)	Prof. Dr. Gernot Marx
German Association for Palliative Medicine (DGP)	Dr. Wiebke Nehls
German Respiratory Society (DGP)*	Prof. Dr. Michael Pfeifer
German Respiratory Society (DGP)*	Prof. Dr. Klaus F. Rabe
German Society of Anaesthesiology and Intensive Care Medicine (DGAI)	PD Dr. Gereon Schälte
German Society of Infectious Diseases (DGI)*	PD Dr. med. Christoph D. Spinner
ARDS network Germany	Prof. Dr. Steffen Weber-Carstens
German Society of Nephrology (DGfN)	Prof. Dr. Julia Weinmann-Menke
German Respiratory Society (DGP)*	Prof. Dr. Tobias Welte
German Society of Anaesthesiology and Intensive Care Medicine (DGAI)	PD Dr. Martin Welper
German Respiratory Society (DGP)*	PD Dr. Michael Westhoff

Guideline contributors and delegates of the medical societies are presented in alphabetic order

*Guideline leading medical society

**Table 4:** Contributors of the COVID-19 evidence ecosystem (CEOsys)

Member	Institution
Marike Andreas	Department I of Internal Medicine, University of Cologne
Kelly Ansems	Department for Surgical Intensive Medicine and Intermediate Care, University Hospital RWTH Aachen
Prof. Dr. Gerhild Becker	Department of Palliative Care, University Medical Center Freiburg
Marie Becker	Department I of Internal Medicine, University of Cologne
Prof. Carina Benstoem	Department for Surgical Intensive Medicine and Intermediate Care, University Hospital RWTH Aachen
PD Dr. Christopher Böhlke	Department of Palliative Care, University Medical Center Freiburg
Karolina Dahms, MSc	Department for Surgical Intensive Medicine and Intermediate Care, University Hospital RWTH Aachen
Elena Dorando	Department I of Internal Medicine, University of Cologne
Dr. Falk Fichtner	Department of Anesthesiology and Intensive Care Medicine, University Hospital Leipzig
Anna-Lena Fischer	Department of Anesthesiology and Intensive Care Medicine, University Hospital Leipzig
Mirko Griesel	Department of Anesthesiology and Intensive Care Medicine, University Hospital Leipzig
Dr. Felicitas Grundeis	Department of Anesthesiology and Intensive Care Medicine, University Hospital Leipzig
Caroline Hirsch	Department I of Internal Medicine, University of Cologne
Claire Iannizzi	Department I of Internal Medicine, University of Cologne
Dr. Karoline Kley	Department of Anesthesiology and Intensive Care Medicine, University Hospital Leipzig
Marco Kopp	Department I of Internal Medicine, University of Cologne
Dr. Andre Kramer	Department of Anesthesiology and Intensive Care Medicine, University Hospital Leipzig
Prof. Dr. Peter Kranke	Department of Anesthesiology, Intensive Care Medicine and Pain Therapy (KAIS), University Hospital Würzburg
Nina Kreuzberger	Department I of Internal Medicine, University of Cologne
PD Dr. Sven Laudi	Department of Anesthesiology and Intensive Care Medicine, University Hospital Leipzig
Prof. Dr. Jörg Meerpohl	Institute for Evidence in Medicine, University Hospital Freiburg
Maria-Inti Metzendorf	Institute of General Practice, Medical Faculty of the Heinrich-Heine University, Düsseldorf
Prof. Dr. Patrick Meybohm	Department of Anesthesiology, Intensive Care Medicine and Pain Therapy (KAIS), University Hospital Würzburg
Dr. Agata Mikolajewska	Department of Infectious Diseases and Respiratory Medicine, Charité-Universitätsmedizin Berlin, Berlin, Germany
Ina Monsef	Department I of Internal Medicine, University of Cologne
Dr. Anika Müller	Association of the Scientific Medical Societies in Germany (AWMF)
Dr. Monika Nothacker	Association of the Scientific Medical Societies in Germany (AWMF)
Oliver Peim	Department of Anesthesiology and Intensive Care Medicine, University Hospital Leipzig
Vanessa Piechotta	Department I of Internal Medicine, University of Cologne
Maria Popp	Department of Anesthesiology, Intensive Care Medicine and Pain Therapy (KAIS), University Hospital Würzburg
PD Dr. Christoph Schmaderer	Department of Nephrology, Technical University of Munich
Dr. Benedikt Schmid	Department of Anesthesiology, Intensive Care Medicine and Pain Therapy (KAIS), University Hospital Würzburg
Dr. Christine Schmucker	Institute for Evidence in Medicine, University Hospital Freiburg
Prof. Dr. Nicole Skoetz	Department I of Internal Medicine, University of Cologne
Dr. Miriam Stegemann	Department of Infectious Diseases and Respiratory Medicine, Charité-Universitätsmedizin Berlin, Berlin, Germany
Julia Kristin Ströhlein	Department I of Internal Medicine, University of Cologne
Dr. Volker Thieme	Department of Anesthesiology and Intensive Care Medicine, University Hospital Leipzig
Carina Wagner	Department I of Internal Medicine, University of Cologne
Julia Wallqvist	Department for Surgical Intensive Medicine and Intermeditate Care, University Hospital RWTH Aachen
Dr. Stefanie Weibel	Department of Anesthesiology, Intensive Care Medicine and Pain Therapy (KAIS), University Hospital Würzburg

Contributors of the CEOsys project are presented in alphabetic order

## References

[1] World Health Organization (WHO) Handbook for guideline development 2nd edition. 2021. https://www.who.int/publications/guidelines/handbook_2nd_ed.pdf?ua=1. (Accessed 18 May 2021).

[2] Moberg J, Oxman AD, Rosenbaum S (2018). The GRADE Evidence to Decision (EtD) framework for health system and public health decisions. Health Res Policy Syst.

[3] AWMF. S3 Leitlinie Empfehlungen zur stationären Therapie von Patienten mit COVID-19 (Registernummer 113-001). Assoc Scient Med Soc Germany (AWMF). https://www.awmf.org/leitlinien/detail/ll/113-001.html. (Accessed 10 May 2021).

[4] Kluge S, Janssens U, Welte T (2020). Recommendations for critically ill patients with COVID-19. Med Klin Intensivmed Notfmed.

[5] Kluge S, Janssens U, Spinner CD (2021). Clinical practice guideline: recommendations on inpatient treatment of patients with COVID-19. Dtsch Arztebl Int.

[6] World Health Organization (WHO) - Working Group on the Clinical_Characterisation and Management of Covid-infection (2020). A minimal common outcome measure set for COVID-19 clinical research. Lancet Infect Dis.

[7] MagicApp. MAGIC Foundation. https://app.magicapp.org. (Accessed 20 May 2021).

[8] Association of the Scientific Medical Societies in Germany (AWMF). AWMF guidance manual and rules for guideline development, 2nd Edition 2020. https://www.awmf.org/leitlinien/awmf-regelwerk/awmf-publikationen-zu-leitlinien/leitlinien-manual.html. (Accessed 20 May 2021).

[9] Horby P, Lim WS, Emberson JR (2021). Dexamethasone in hospitalized patients with Covid-19. N Engl J Med.

[10] Tomazini BM, Maia IS, Cavalcanti AB (2020). Effect of dexamethasone on days alive and ventilator-free in patients with moderate or severe acute respiratory distress syndrome and COVID-19: the CoDEX randomized clinical trial. JAMA.

[11] Edalatifard M, Akhtari M, Salehi M (2020). Intravenous methylprednisolone pulse as a treatment for hospitalised severe COVID-19 patients: results from a randomised controlled clinical trial. Eur Respir J.

[12] Dequin PF, Heming N, Meziani F (2020). Effect of hydrocortisone on 21-day mortality or respiratory support among critically Ill patients with COVID-19: a randomized clinical trial. JAMA.

[13] Jeronimo CMP, Farias MEL, Val FFA (2021). Methylprednisolone as adjunctive therapy for patients hospitalized with coronavirus disease 2019 (COVID-19; Metcovid): a randomized, double-blind, phase IIb Placebo-controlled Trial. Clin Infect Dis.

[14] Angus DC, Derde L, Al-Beidh F (2020). Effect of hydrocortisone on mortality and organ support in patients with severe COVID-19: the REMAP-CAP COVID-19 corticosteroid domain randomized clinical trial. JAMA.

[15] Hermine O, Mariette X, Tharaux PL (2021). Effect of tocilizumab vs usual care in adults hospitalized with COVID-19 and moderate or severe pneumonia: a randomized clinical trial. JAMA Intern Med.

[16] Salama C, Han J, Yau L (2021). Tocilizumab in patients hospitalized with Covid-19 pneumonia. N Engl J Med.

[17] Salvarani C, Dolci G, Massari M (2021). Effect of tocilizumab vs standard care on clinical worsening in patients hospitalized with COVID-19 pneumonia: a randomized clinical trial. JAMA Intern Med.

[18] Stone JH, Frigault MJ, Serling-Boyd NJ (2020). Efficacy of tocilizumab in patients hospitalized with Covid-19. N Engl J Med.

[19] Veiga VC, Prats J, Farias DLC (2021). Effect of tocilizumab on clinical outcomes at 15 days in patients with severe or critical coronavirus disease 2019: randomised controlled trial. BMJ.

[20] Gordon AC, Mouncey PR, Al-Beidh F (2021). Interleukin-6 receptor antagonists in critically Ill patients with Covid-19. N Engl J Med.

[21] Rosas IO, Bräu N, Waters M (2021). Tocilizumab in hospitalized patients with severe Covid-19 pneumonia. N Engl J Med.

[22] Peter W, Horby MC, Staplin N, Spata E (2021). Tocilizumab in patients admitted to hospital with COVID-19 (RECOVERY) a randomised, controlled, open-label, platform trial. Lancet.

[23] Soin AS, Kumar K, Choudhary NS (2021). Tocilizumab plus standard care versus standard care in patients in India with moderate to severe COVID-19-associated cytokine release syndrome (COVINTOC): an open-label, multicentre, randomised, controlled, phase 3 trial. Lancet Respir Med.

[24] Beigel JH, Tomashek KM, Dodd LE (2020). Remdesivir for the treatment of Covid-19 - final report. N Engl J Med.

[25] Pan H, Peto R, Henao-Restrepo AM (2021). Repurposed antiviral drugs for Covid-19 - interim WHO solidarity trial results. N Engl J Med.

[26] Spinner CD, Gottlieb RL, Criner GJ (2020). Effect of remdesivir vs standard care on clinical status at 11 days in patients with moderate COVID-19: a randomized clinical trial. JAMA.

[27] Wang Y, Zhang D, Du G (2020). Remdesivir in adults with severe COVID-19: a randomised, double-blind, placebo-controlled, multicentre trial. Lancet.

[28] Goldman JD, Lye DCB, Hui DS (2020). Remdesivir for 5 or 10 days in patients with severe Covid-19. N Engl J Med.

[29] World Health Organization (WHO). COVID-19 Clinical management Living guidance 2021. https://apps.who.int/iris/rest/bitstreams/1328457/retrieve. (Accessed 20 May 2021).

[30] Simonis A, Theobald SJ, Fätkenheuer G (2020). A comparative analysis of remdesivir and other repurposed antivirals against SARS-CoV-2. EMBO Mol Med.

[31] Li L, Zhang W, Hu Y (2020). Effect of convalescent plasma therapy on time to clinical improvement in patients with severe and life-threatening COVID-19: a randomized clinical trial. JAMA.

[32] Agarwal A, Mukherjee A, Kumar G (2020). Convalescent plasma in the management of moderate covid-19 in adults in India: open label phase II multicentre randomised controlled trial (PLACID Trial). BMJ.

[33] Simonovich VA, Burgos Pratx LD, Scibona P (2021). A randomized trial of convalescent plasma in Covid-19 severe pneumonia. N Engl J Med.

[34] Hamdy Salman O, Ail Mohamed HS (2020). Efficacy and safety of transfusing plasma from COVID-19 survivors to COVID-19 victims with severe illness. A double-blinded controlled preliminary study. Egypt J Anaesth.

[35] Lundgren JD, Grund B, Barkauskas CE (2021). A neutralizing monoclonal antibody for hospitalized patients with Covid-19. N Engl J Med.

[36] Weinreich DM, Sivapalasingam S, Norton T (2021). REGN-COV2, a neutralizing antibody cocktail, in outpatients with Covid-19. N Engl J Med.

[37] Chen P, Nirula A, Heller B (2021). SARS-CoV-2 neutralizing antibody LY-CoV555 in outpatients with Covid-19. N Engl J Med.

[38] Gottlieb RL, Nirula A, Chen P (2021). Effect of bamlanivimab as monotherapy or in combination with etesevimab on viral load in patients with mild to moderate COVID-19: a randomized clinical trial. JAMA.

[39] U.S. Food and Drug Administration (FDA). Fact sheet for health care providers emergency use authorization (EUA) of bamlanivimab and etesevimab. www.fda.gov/media/145802/download. (Accessed 12 May 2021).

[40] Association of the Scientific Medical Societies in Germany (AWMF). S3-Leitlinie prophylaxe der venösen thromboembolie (VTE). https://www.awmf.org/leitlinien/detail/ll/003-001.html. (Accessed 12 May 2021).

[41] Wichmann D, Sperhake JP, Lütgehetmann M (2020). Autopsy findings and venous thromboembolism in patients with COVID-19: a prospective cohort study. Ann Intern Med.

[42] Langer F, Kluge S, Klamroth R (2020). Coagulopathy in COVID-19 and its implication for safe and efficacious thromboprophylaxis. Hamostaseologie.

[43] Cohen AT, Davidson BL, Gallus AS (2006). Efficacy and safety of fondaparinux for the prevention of venous thromboembolism in older acute medical patients: randomised placebo controlled trial. BMJ.

[44] Samama MM, Cohen AT, Darmon JY (1999). A comparison of enoxaparin with placebo for the prevention of venous thromboembolism in acutely ill medical patients. Prophylaxis in medical patients with enoxaparin study group. N Engl J Med.

[45] Leizorovicz A, Cohen AT, Turpie AG (2004). Randomized, placebo-controlled trial of dalteparin for the prevention of venous thromboembolism in acutely ill medical patients. Circulation.

[46] Patell R, Chiasakul T, Bauer E (2021). Pharmacologic thromboprophylaxis and thrombosis in hospitalized patients with COVID-19: a pooled analysis. Thromb Haemost.

[47] Middeldorp S, Coppens M, van Haaps TF (2020). Incidence of venous thromboembolism in hospitalized patients with COVID-19. J Thromb Haemost.

[48] Klok FA, Kruip M, van der Meer NJM (2020). Confirmation of the high cumulative incidence of thrombotic complications in critically ill ICU patients with COVID-19: an updated analysis. Thromb Res.

[49] Sadeghipour P, Talasaz AH, Rashidi F (2021). Effect of intermediate-dose vs. standard-dose prophylactic anticoagulation on thrombotic events, extracorporeal membrane oxygenation treatment, or mortality among patients with COVID-19 admitted to the intensive care unit: the INSPIRATION randomized clinical trial. JAMA.

[50] Lawler PR, Goligher EC, Berger JS (2021). Therapeutic anticoagulation in non-critically Ill patients with Covid-19. medrXiv.

[51] Abaleke E, Abbas M, Abbasi S (2021). Azithromycin in patients admitted to hospital with COVID-19 (RECOVERY): a randomised, controlled, open-label, platform trial. Lancet.

[52] Furtado RHM, Berwanger O, Fonseca HA (2020). Azithromycin in addition to standard of care versus standard of care alone in the treatment of patients admitted to the hospital with severe COVID-19 in Brazil (COALITION II): a randomised clinical trial. Lancet.

[53] Cavalcanti AB, Zampieri FG, Rosa RG (2020). Hydroxychloroquine with or without azithromycin in mild-to-moderate Covid-19. N Engl J Med.

[54] Sekhavati E, Jafari F, SeyedAlinaghi S (2020). Safety and effectiveness of azithromycin in patients with COVID-19: an open-label randomised trial. Int J Antimicrob Agents.

[55] Dalhoff K, Abele-Horn M, Andreas S (2018). Epidemiology, diagnosis and treatment of adult patients with nosocomial pneumonia - update 2017. Pneumologie.

[56] Ewig S, Höffken G, Kern WV (2016). Management of adult community-acquired pneumonia and prevention - Update 2016. Pneumologie.

[57] Krolewiecki A, Lifschitz A, Moragas M (2021). Antiviral effect of high-dose ivermectin in adults with COVID-19: a pilot randomised, controlled, open label. Multicentre Trial.

[58] Carlos C, Aina C, Andres Blanco-Di M (2021). The effect of early treatment with ivermectin on viral load, symptoms and humoral response in patients with mild COVID-19: a pilot, double-blind, placebo-controlled, randomized clinical trial. Res Square.

[59] Ravikirti RR, Pattadar C (2021). Ivermectin as a potential treatment for mild to moderate COVID-19 – A double blind randomized placebo-controlled trial. medrXiv.

[60] Ahmed S, Karim MM, Ross AG (2021). A five-day course of ivermectin for the treatment of COVID-19 may reduce the duration of illness. Int J Infect Dis.

[61] Jermain B, Hanafin PO, Cao Y (2020). Development of a minimal physiologically-based pharmacokinetic model to simulate lung exposure in humans following oral administration of ivermectin for COVID-19 drug repurposing. J Pharm Sci.

[62] Entrenas Castillo M, Entrenas Costa LM, Vaquero Barrios JM (2020). Effect of calcifediol treatment and best available therapy versus best available therapy on intensive care unit admission and mortality among patients hospitalized for COVID-19: a pilot randomized clinical study. J Steroid Biochem Mol Biol.

[63] Ginde AA, Brower RG, Caterino JM (2019). Early high-dose vitamin D(3) for critically Ill, vitamin D-deficient patients. N Engl J Med.

[64] Murai IH, Fernandes AL, Sales LP (2021). Effect of a single high dose of vitamin D3 on hospital length of stay in patients with moderate to severe COVID-19: a randomized clinical trial. JAMA.

[65] Deutsche Gesellschaft für Ernährung E. V. (DGE). Zum Zusammenhang zwischen der Vitamin-D-Zufuhr bzw. dem Vitamin-D-Status und dem Risiko für eine SARS-CoV-2-Infektion sowie der Schwere des Verlaufs einer COVID-19-Erkrankung – ein Überblick über die aktuelle Studienlage (Stand 11. Januar 2021). www.dge.de/uploads/media/DGE_Fachinfo_VitaminD_COVID-19_Stand_Januar_2021.pdf. (Accessed 12 May 2021).

[66] Malin JJ, Suárez I, Priesner V (2020). Remdesivir against COVID-19 and other viral diseases. Clin Microbiol Rev.

[67] Kalil AC, Patterson TF, Mehta AK (2021). Baricitinib plus remdesivir for hospitalized adults with Covid-19. N Engl J Med.

